# Cancer Survivorship Care in the United States at Facilities Accredited by the Commission on Cancer

**DOI:** 10.1001/jamanetworkopen.2024.18736

**Published:** 2024-07-03

**Authors:** Julia Stal, Kimberly A. Miller, Timothy W. Mullett, Judy C. Boughey, Amanda B. Francescatti, Elizabeth Funk, Heidi Nelson, David R. Freyer

**Affiliations:** 1 Department of Population and Public Health Sciences, Keck School of Medicine, University of Southern California, Los Angeles; 2 Cancer Research Program, American College of Surgeons, Chicago, Illinois; 3 Department of Dermatology, Keck School of Medicine, University of Southern California, Los Angeles; 4 USC Norris Comprehensive Cancer Center, Los Angeles, California; 5 Markey Cancer Center, Cancer Prevention and Control Program, University of Kentucky, Lexington; 6 Department of Surgery, College of Medicine, University of Kentucky, Lexington; 7 Commission on Cancer, American College of Surgeons, Chicago, Illinois; 8 Department of Surgery, Mayo Clinic, Rochester, Minnesota; 9 Department of Pediatrics, Keck School of Medicine, University of Southern California, Los Angeles; 10 Department of Medicine, Keck School of Medicine, University of Southern California, Los Angeles; 11 Cancer and Blood Disease Institute, Children’s Hospital Los Angeles, Los Angeles, California

## Abstract

**Importance:**

Since 2021, American College of Surgeons Commission on Cancer (CoC) accreditation standards require providing a survivorship program for patients with adult-onset cancer treated with curative intent. Since more than 70% of all patients with cancer in the US are treated at CoC-accredited facilities, this presents an opportunity for a landscape analysis of survivorship care availability.

**Objective:**

To determine the prevalence, types, and outcomes of cancer survivorship services at CoC-accredited facilities.

**Design, Setting, and Participants:**

This survey study used an anonymous, online, cross-sectional survey conducted from May 4 to 25, 2023. Participants were CoC-accredited facilities in the US representing diverse CoC program categories, institutional characteristics, geographic regions, and practice types. Department of Veterans Affairs cancer programs were excluded due to data usage restrictions. Data were analyzed from July to October 2023.

**Exposure:**

CoC Survivorship Standard 4.8 was released in October 2019 and programs were expected to adhere to the Standard beginning January 1, 2021.

**Main Outcomes and Measures:**

Questions included self-reported survivorship program characteristics, availability of services aligned to CoC Survivorship Standard 4.8, and perceived program impacts. Response frequencies and proportions were determined in aggregate and by CoC program category.

**Results:**

There were 1400 eligible programs, and 384 programs participated (27.4% response rate). All regions and eligible program categories were represented, and most had analytic caseloads of 500 to 4999 patients in 2021. Most survivorship program personnel included nurses (334 programs [87.0%]) and social workers (278 programs [72.4%]), while physical (180 programs [46.9%]) and occupational (87 programs [22.7%]) therapists were less common. Services most endorsed as available for all survivors were screening for new cancers (330 programs [87.5%]), nutritional counseling (325 programs [85.3%]), and referrals to specialists (320 programs [84.7%]), while treatment summaries (242 programs [64.7%]), and survivorship care plans (173 programs [43.0%]), sexual health (217 programs [57.3%]), and fertility (214 programs [56.9%]) were less common. Survivorship services were usually delivered by cancer treatment teams (243 programs [63.3%]) rather than specialized survivorship clinics (120 programs [31.3%]). For resources needed, additional advanced practice clinicians with dedicated survivorship effort (205 programs [53.4%]) and electronic health record enhancements (185 programs [48.2%]) were most endorsed. Lack of referrals and low patient awareness were endorsed as the primary barriers. A total of 335 programs (87.2%) agreed that Survivorship Standard 4.8 helped advance their programs.

**Conclusions and Relevance:**

These findings of this survey study of CoC-accredited programs establish a benchmark for survivorship care delivery in the US, identify gaps in specific services and opportunities for intervention, contribute to longitudinal reevaluation for tracking progress nationally, and suggest the value of survivorship care standards.

## Key Points

**Question:**

What are the prevalence and types of survivorship services currently available to survivors of adult-onset cancer in the United States?

**Findings:**

In this survey study of 384 facilities accredited by the American College of Surgeons Commission on Cancer, sexual health and fertility services were provided less commonly than other services, and survivorship services were usually delivered by cancer treatment teams rather than specialized survivorship clinics. More than 80% of institutions perceived their survivorship services as beneficial but estimated a minority of survivors received them; a lack of referrals and low patient awareness were endorsed as primary barriers.

**Meaning:**

These findings establish a national benchmark for survivorship care delivery, identify gaps in specific services and opportunities for intervention, and contribute to longitudinal reevaluation for tracking progress.

## Teaser

This survey study examines the prevalence, types, and outcomes of cancer survivorship services at US cancer care facilities accredited by the American College of Surgeons Commission on Cancer.

## Introduction

In the US, 5-year relative survival after diagnosis with cancer now exceeds 68%.^1^ In 2022, there were more than 18 million survivors of cancer in the US population, representing 5.4% of the population, and this number is projected to increase to 22.5 million by 2032.^2,3,4^ Most of these individuals are survivors of adult-onset cancer, and most will remain in remission and experience years of additional life.^5^ Unfortunately, survivors of adult-onset cancer may develop a variety of clinically significant late effects of treatment causing premature mortality, chronic illness, and lower quality of life.^6,7,8,9,10,11,12^ Comprehensive survivorship care can facilitate the early recognition, optimal management, and secondary prevention of many late effects, as well as delivery of health information and psychosocial support.^13^ For these reasons, the National Comprehensive Cancer Network and American Society of Clinical Oncology recommend routine provision of survivorship care after treatment of adult-onset cancer.^14,15^

The American College of Surgeons (ACS) Commission on Cancer (CoC) accredits institutions that meet CoC standards addressing key components of quality cancer care.^16,17^ Currently, there are more than 1400 CoC-accredited cancer programs in the US, which include both academic and community-based practices and treat more than 70% of all newly diagnosed patients.^18^ Since 2015, CoC standards have incorporated survivorship care as an accreditation criterion. Initially, CoC Survivorship Standard 3.3 set targets for delivery of survivorship care plans to patients.^19,20^ In 2021, this was replaced by the current CoC Survivorship Standard 4.8, which requires institutions to offer a survivorship program for patients treated with curative intent.^16,21^ Expectations for Survivorship Standard 4.8 include appointing a survivorship program coordinator, developing a multidisciplinary survivorship program team, and determining a list of services, offered on site or by referral, that address the needs of survivors of cancer. Documentation of at least 3 services and their impact is required annually, with enhancement of these and additional services expected over time.^16,21^

Despite survivorship being recognized as part of the cancer care continuum, limited research has examined the availability and types of services for survivors of adult-onset cancer. This is in contrast to the field of pediatric oncology, in which guideline-directed survivorship care is routinely provided in dedicated clinics whose prevalence and characteristics are well-documented.^14,15,22,23,24,25^ Given the ACS CoC’s national presence and inclusion of survivorship care as an accreditation standard, this consortium of institutions offers a unique opportunity for examining survivorship services for patients with adult-onset cancer. The purpose of this study was to characterize the prevalence and types of survivorship services for patients with adult-onset cancer among CoC-accredited programs. Understanding the landscape of these services in the US will provide a national benchmark and contribute to identifying gaps, strengths, and interventional opportunities for improving survivorship care for this large and growing population.

## Methods

This survey study was deemed exempt from review by the Advarra Institutional Review Board because the study posed no more than minimal risk and responses were anonymous. All survey respondents provided informed consent. This study followed relevant best practices of the American Association for Public Opinion Research (AAPOR) reporting guideline.^26^

### Study Design, Setting, and Participating Institutions

An online, cross-sectional survey of ACS CoC-accredited programs (eAppendix 1 in Supplement 1) was administered from May 4 to May 25, 2023, a survey window congruent with ACS practice. The cohort comprised facilities belonging to 1 of 8 predefined membership categories: Academic Comprehensive Cancer Programs, Comprehensive Community Cancer Programs, Community Cancer Programs, Free-Standing Cancer Center Programs, Hospital Associate Cancer Programs, Integrated Network Cancer Programs, National Cancer Institute (NCI)–Designated Comprehensive Cancer Programs, and NCI-Designated Networks (eAppendix 1 in Supplement 1). Excluded categories were pediatric cancer programs (due to study focus on adult-onset cancer) and Department of Veterans Affairs cancer programs (due to data usage protections). No incentives were provided. The survey was available in English only. Data were collected and managed using REDCap software (REDCap Consortium)^27,28^ hosted by the ACS.

### Recruitment and Survey Procedures

Eligible institutions were invited to participate via an email sent to their ACS cancer program administrator (CPA) of record. Email invitations described the study purpose, significance, and survey length (15-20 minutes). CPAs received an information sheet and instruction to engage other institutional personnel as appropriate, including the cancer committee chair, cancer liaison physician, survivorship program coordinator, and certified tumor registrar (eAppendix 1 in Supplement 1). A reminder email was sent 1 week following the initial invitation via REDCap. Only 1 survey per institution was accepted. All responses were anonymous and deidentified.

### Survey Development and Content

Survey items were developed iteratively by the study team. To calibrate the survey draft, 12 representative ACS CoC programs were invited to provide deidentified feedback, which led to minor adjustments.

The survey solicited information on facility characteristics and care components recommended in CoC Survivorship Standard 4.8.^16^ Accordingly, a survivorship program was defined as meeting the needs of patients with adult-onset cancer treated with curative intent; a survivor of cancer was defined as someone who has completed the acute phase of conventional therapy (ie, cytotoxic chemotherapy, radiation therapy, cancer-directed surgery) but might still be receiving an extended course or chronic phase of noncytotoxic maintenance therapy (eg, aromatase inhibitors or immunomodulators). Items used a forced-choice format; where applicable, respondents endorsed 1 or more response options and could provide free-response information (eAppendix 2 in Supplement 1).

Item categories were institutional characteristics, survivorship program team, survivorship program services, specialized survivorship clinic, survivorship program components, resource needs, and program perceptions. Institutional characteristics included ACS institutional category, analytic case load for calendar year 2021, geographic region, and respondent role. Survivorship program team characteristics were team members responsible for implementing Survivorship Standard 4.8, such as nurses, social workers, coordinators, advanced practice clinicians (APCs), nutritionists, physicians, and physical and occupational therapists.^16^ Survivorship program services were defined as available survivorship services aligned to Survivorship Standard 4.8.^16^ Services were categorized as care delivery services (ie, specialist referrals, treatment summaries, survivorship care plans), clinical services (ie, screening for new or recurrent cancers, nutritional services, rehabilitation services, cancer genetics counseling, sexual health services, fertility consultation and management), and psychosocial services (ie, psychological and psychiatric services, financial counseling, patient support groups or seminars, physical activity and fitness programs). Services could be provided on site at the accredited facility or affiliate or through referral to an unaffiliated, external facility. For each service, respondents indicated its availability for all or only a subset of survivors. Specialized survivorship clinics were defined as those providing survivorship care to all patients with adult-onset cancer or selected subsets (eg, by cancer type, treatment modality, age). Survivorship program components included regular team meetings, clinical team composition, survivorship website or marketing, philanthropic support, survivorship clinic or clinics, survivorship physicians, institutional funding, informatics, office or research support staff, and survivorship program budget or cost center. Resource needs were assessed by asking institutions to select the 5 most important resources not already available that would help the survivorship program achieve its goals. Options included survivorship physicians, APCs, office or research support staff, designated clinic space, increased referrals, enhanced electronic health record system, informatics, greater program recognition, institutional funding, and philanthropic support. Program perceptions were assessed as institutional perspectives regarding whether their survivorship program existed before introduction of Survivorship Standard 4.8, whether this standard helped advance survivorship care at their institution, what proportion of eligible patients receive survivorship care, and barriers to and perceived benefits of survivorship care.

### Statistical Analysis

Programs that completed more than 50% of the survey were included in the analytic sample. Analyses included frequencies and contingency tables to characterize responses in aggregate and across CoC program categories. Analysis was performed using Stata software version 15 (StataCorp). Data were analyzed from July 2023 to October 2023.

## Results

### Study Sample

Derivation of the analytic sample is summarized in the eFigure in Supplement 1. The survey was sent to 1400 eligible programs, of which 1353 were reached. Overall, 1022 CPAs were responsible for 1 institution each, while 116 CPAs were responsible for more than 1 institution each and received a corresponding number of unique links (range, 2-10 per CPA). Ultimately, 439 responses were received (380 fully completed, 59 partially completed). After data cleaning, 55 partially completed responses were dropped and 4 were retained. The final analytic sample comprised 384 programs (response rate, 27.4%) from across all 8 eligible categories in similar proportions to CoC programs overall (Table 1).

**Table 1.** Institutional Characteristics

Characteristic	Total, No. (%)^b^	CoC program category, No. (%)^a^							
		Comprehensive Community Cancer Program	Community Cancer Program	Integrated Cancer Network Program	Academic Comprehensive Cancer Program	Hospital Associate Cancer Program	NCI-Designated Comprehensive Cancer Program	Free-Standing Cancer Center Program	NCI-Designated Network
Total^c^	371 (96.6)	122 (32.9)	84 (22.6)	65 (17.5)	48 (12.9)	29 (7.8)	19 (5.1)	3 (0.8)	1 (0.3)
Analytic load for calendar year 2021, patients, No.									
0-99	8 (2.2)	1 (0.8)	2 (2.5)	2 (3.1)	0	3 (12.6)	0	0	0
100-249	31 (8.6)	4 (3.4)	14 (17.2)	1 (1.6)	2 (4.4)	8 (33.3)	1 (5.2)	1 (33.3)	0
250-499	52 (14.4)	10 (8.4)	35 (43.2)	3 (4.7)	0	2 (8.3)	0	1 (33.3)	0
500-999	80 (22.1)	44 (37.0)	17 (21.0)	13 (20.3)	3 (6.7)	2 (8.3)	0	0	0
1000-4999	161 (44.5)	54 (45.4)	11 (13.6)	37 (57.8)	37 (82.2)	8 (33.3)	9 (47.4)	1 (33.3)	1 (100)
≥5000	30 (8.3)	6 (5.0)	2 (2.5)	8 (12.5)	3 (6.7)	1 (4.2)	9 (47.4)	0	0
Geographic region									
East North Central^d^	88 (23.2)	32 (26.2)	17 (20.2)	17 (26.2)	9 (19.2)	8 (28.6)	1 (5.3)	1 (33.3)	0
South Atlantic^e^	58 (15.3)	17 (13.9)	17 (20.2)	8 (12.3)	7 (14.9)	7 (25.0)	0	0	1 (100)
Middle Atlantic^f^	55 (14.5)	15 (12.3)	5 (6.0)	13 (20.0)	13 (27.7)	4 (14.3)	5 (26.3)	0	0
Pacific^g^	44 (11.6)	12 (9.8)	13 (15.5)	4 (6.2)	4 (8.5)	2 (7.1)	7 (36.8)	0	0
West South Central^h^	38 (10.0)	16 (13.1)	8 (9.5)	2 (3.1)	6 (12.8)	0	2 (10.5)	2 (66.7)	0
East South Central^i^	34 (9.0)	12 (9.8)	9 (10.7)	11 (16.9)	1 (2.1)	0	0	0	0
New England^j^	24 (6.3)	7 (5.7)	7 (8.3)	4 (6.2)	3 (6.4)	1 (3.6)	1 (5.3)	0	0
West North Central^k^	21 (5.5)	4 (3.3)	6 (7.1)	2 (3.1)	4 (8.5)	3 (10.7)	1 (5.3)	0	0
Mountain^l^	18 (4.7)	7 (5.7)	2 (2.4)	4 (6.2)	0	3 (10.7)	2 (10.5)	0	0
Role of person completing survey^m^									
Cancer program administrator	187 (48.7)	67 (54.9)	44 (52.4)	40 (61.5)	20 (41.7)	10 (34.5)	3 (15.8)	0	1 (100)
Survivorship program coordinator	141 (36.7)	38 (31.2)	26 (31.0)	23 (35.4)	19 (39.6)	14 (48.3)	11 (57.9)	3 (100)	0
Certified tumor registrar	31 (8.1)	12 (9.8)	5 (6.0)	5 (7.7)	7 (14.6)	1 (3.5)	1 (5.3)	0	0
Cancer committee chair	11 (2.9)	1 (0.8)	5 (6.0)	0	2 (4.2)	2 (6.9)	1 (5.3)	0	0
Cancer liaison physician	1 (0.3)	0	0	0	1 (2.1)	0	0	0	0
Other^n^	48 (12.5)	16 (13.1)	15 (17.9)	5 (7.7)	5 (10.4)	2 (6.9)	3 (15.8)	0	0

Abbreviations: CoC, Commission on Cancer; NCI, National Cancer Institute.

^a^ Percentages are given for columns.

^b^ The denominator for this column is 384. Total values may not sum to 384 due to item missingness.

^c^ For comparison, CoC program category proportions of record^18^ are as follows: 38% are Comprehensive Community Cancer Programs, 26% are Community Cancer Programs, 14% are Integrated Cancer Network Programs, 13% are Academic Comprehensive Cancer Programs, 1% are Hospital Associate Cancer Programs, 3% are NCI-Designated Comprehensive Cancer Programs, 1% are Free-Standing Cancer Center Programs, and 1% are NCI-Designated Networks.

^d^ Includes Illinois, Indiana, Michigan, Ohio, and Wisconsin.

^e^ Includes the District of Columbia, Delaware, Florida, Georgia, Maryland, North Carolina, South Carolina, Virginia, and West Virginia.

^f^ Includes New Jersey, New York, and Pennsylvania.

^g^ Includes Alaska, California, Hawaii, Oregon, and Washington.

^h^ Includes Arkansas, Louisiana, Oklahoma, and Texas.

^i^ Includes Alabama, Kentucky, Mississippi, and Tennessee.

^j^ Includes Connecticut, Massachusetts, Maine, New Hampshire, Rhode Island, and Vermont.

^k^ Includes Iowa, Kansas, Minnesota, Missouri, North Dakota, Nebraska, and South Dakota.

^l^ Includes Arizona, Colorado, Idaho, Montana, New Mexico, Nevada, Utah, and Wyoming.

^m^ Participants could select more than 1 response. Percentages are given for rows.

^n^ Includes accreditation manager, accreditation coordinator, cancer data coordinator, cancer quality coordinator, clinical supervisor, director of cancer care services, director of quality and safety, director of patient and family services, medical director, nurse practitioner, nurse manager, nurse navigator, physician center manager, project coordinator, quality improvement coordinator, social work manager, survivorship cocoordinator, and survivorship physician assistant.

### Institutional Characteristics

Table 1 summarizes institutional characteristics in aggregate and by CoC program category. Of the 384 total programs, 371 (96.6%) reported their CoC program category. Of these, more than half were Comprehensive Community Cancer Programs (122 programs [32.9%]) or Community Cancer Programs (84 programs [22.6%]). Two-thirds of programs reported analytic caseloads of 500 to 999 patients (80 programs [22.1%]) or 1000 to 4999 patients (161 programs [44.5%]) for 2021. All geographic regions were represented, the largest being East North Central (88 programs [23.2%]). Most surveys were completed by CPAs (187 surveys [48.7%]) or survivorship program coordinators (141 surveys [36.7%]).

### Survivorship Program Team Members

**Figure 1.**
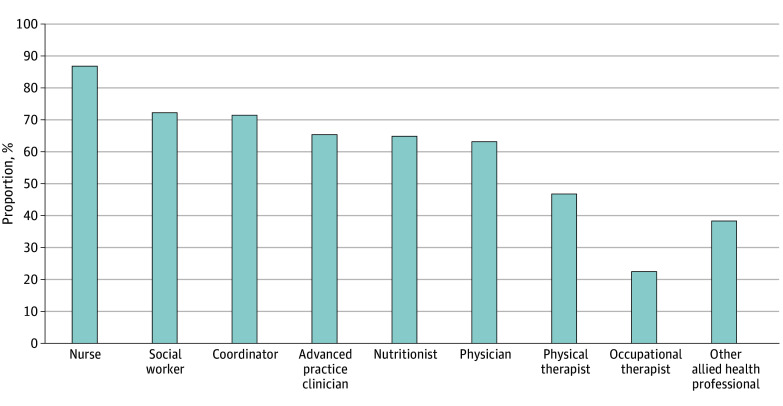
Survivorship Program Team Members Bars indicate the percentage of respondents in aggregate that endorsed each role as a member of their survivorship program team. Respondents selected all that applied. The total number of responses was 384. Other members reported by 148 programs were acupuncturist, administrator, art and music therapist, massage therapist, cancer center manager, cancer program coordinator, cancer program associate, cancer registrar, case manager, certified community health worker, chaplain, clinical trial nurse, community partner, director of rehabilitation, education coordinator, exercise physiologist, financial counselor or navigator, integrative health specialist, life coach, lymphedema therapist, medical assistant, mindfulness counselor, oncology counselor, palliative care, patient navigator, pharmacist, psychology or behavioral health counselor, radiation therapist, researcher, respiratory therapist, sexual health counselor, speech-language pathologist, speech therapist, spiritual care, tobacco cessation coordinator, tumor registry, wellness specialist, and yoga instructor. Team members by program category are provided in eTable 1 in Supplement 1.

Figure 1 summarizes team members. Most programs identified nurses (334 programs [87.0%]), followed by social workers (278 programs [72.4%]), program coordinators (275 programs [71.6%]), APCs (252 programs [65.6%]), nutritionists (250 programs [65.1%]), and physicians (243 programs [63.3%]). Less than half of the included programs reported physical (180 programs [46.9%]) or occupational (87 programs [22.7%]) therapists (Figure 1). Free responses highlighted a wide variety of other roles. Survivorship team composition was relatively consistent across CoC program categories (eTable 1 in Supplement 1), although Integrated Cancer Network, Academic Comprehensive Cancer, and NCI-Designated Cancer programs reported greater representation of physicians and APCs.

### Survivorship Program Services

**Figure 2.**
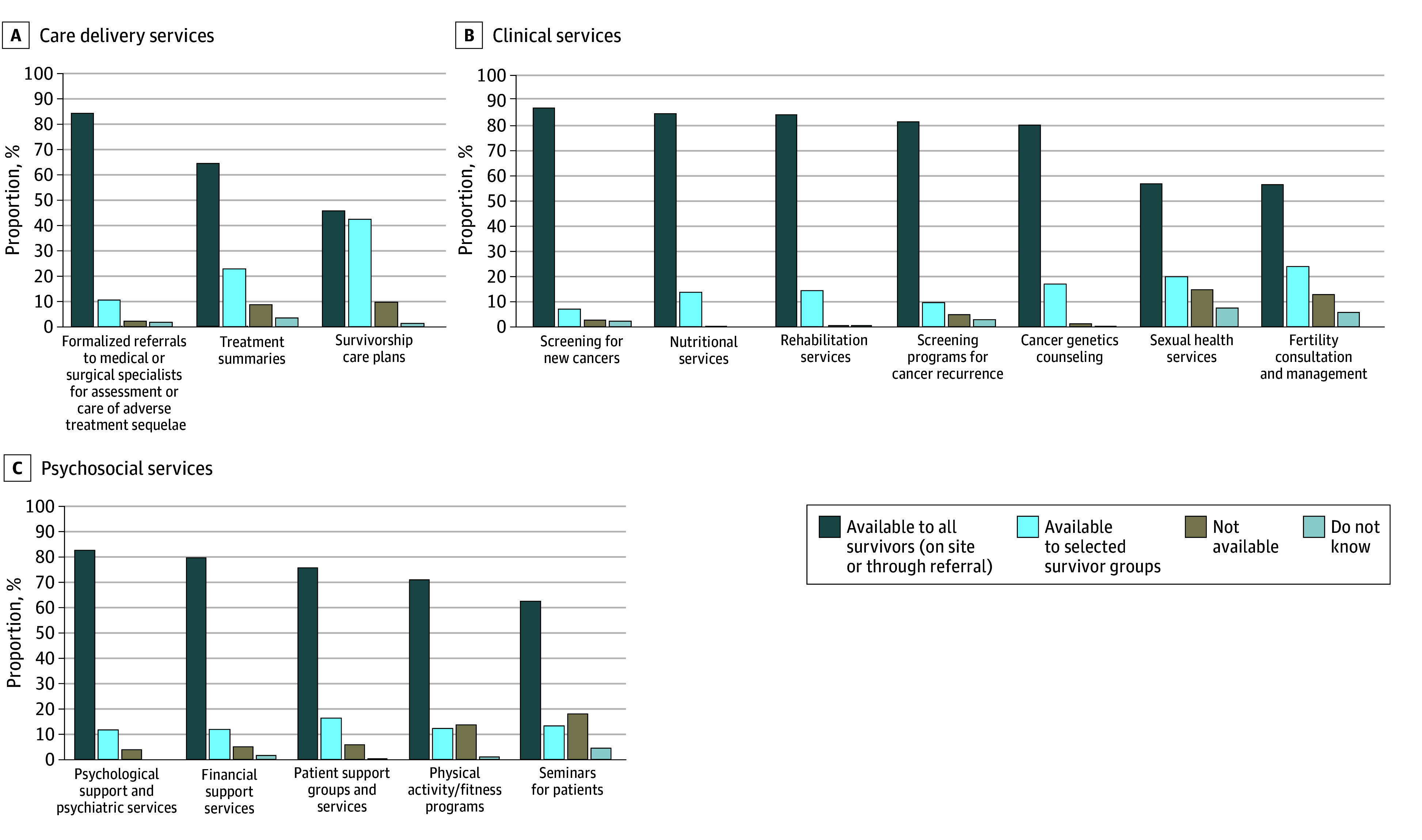
Survivorship Services Currently Available Bars indicate the percentage of respondents in aggregate that endorsed each service as currently available in their survivorship program. Respondents selected all that applied. The total number of responses for each service ranged from 374 to 382. Other services reported by 36 institutions were acupuncture, advanced care planning, art therapy, bone health clinic, cancer cognitive clinic through neurology, cancer mindfulness, cancer rehabilitation services, chaplain or pastoral care and spiritual support, child life specialist, clinical trials, community resources, cooking classes, expressive arts through medicine, hearing and audiology, integrative health classes, integrative medicine, lifelong movement program, lifestyle medicine, massage, mental wellness program, mindfulness meditation class, music therapy, nurse navigation, oncology dietitian, online nutrition class, palliative services, pelvic floor therapy, recorded lectures, physical medicine and rehabilitation, precision genomics program, reiki therapy, return to work program, 7-week cancer survivorship program, smoking cessation, spirituality classes, wellness classes, and yoga therapy.

Figure 2 summarizes available survivorship program services. Most programs reported offering multiple services. Of the 15 services queried, 11 were individually endorsed by more than 90% of programs as available either to all survivors or certain subsets; 8 services were endorsed by more than 80% of programs as available for all survivors. Among care delivery services (Figure 2A), formal specialist referrals for managing late effects were available to all or some survivors at 361 institutions (95.6%). Treatment summaries or survivorship care plans were offered at approximately 90% of programs, although treatment summaries were more widely available (242 programs [64.7%]) than survivorship care plans (173 programs [46.0%]). Among clinical services (Figure 2B), screening for new and/or recurrent cancers (330 programs [87.5%]), nutritional services (325 programs [85.3%]), rehabilitation (319 programs [84.6%]), and genetics counseling (305 programs [80.7%]) were frequently available for all survivors. By contrast, sexual health and fertility services were available at less than 60% of programs for all survivors, and instead were either offered to certain patient subsets (sexual health: 76 programs [20.1%]; fertility: 91 programs [24.2%]) or were completely unavailable (sexual health: 57 programs [15.0%]; fertility: 49 programs [13.0%]). Among psychosocial services (Figure 2C), psychological and psychiatric services, financial counseling, and patient support groups were endorsed by more than 90% of programs as available to all or some survivors, but patient support groups were more often limited to specific survivor populations (64 programs [16.8%]) than were other psychosocial services. Physical activity and fitness programs and patient seminars were most frequently endorsed as not available (physical activity and fitness: 53 programs [14.1%]; patient seminars: 70 programs [18.5%]). Free responses indicated a wide variety of other survivorship services offered.

### Specialized Survivorship Clinics, Program Components, and Resources Needed

#### Specialized Survivorship Clinics

As summarized in eTable 2 in Supplement 1, 120 programs (31.3%) endorsed offering a specialized survivorship clinic. Of these, 58 programs (48.3%) offered clinics for all survivors and 57 programs (47.5%) for certain subsets (with some offering both). Comprehensive Community Cancer Programs reported the highest percentage of clinics serving all survivors (17 programs [60.7%]), while Hospital Associate Cancer Programs (6 programs [60.0%]) and NCI-Designated Cancer Centers (8 programs [57.1%]) more frequently reported clinics serving subsets of patients.

#### Survivorship Program Components

**Figure 3.**
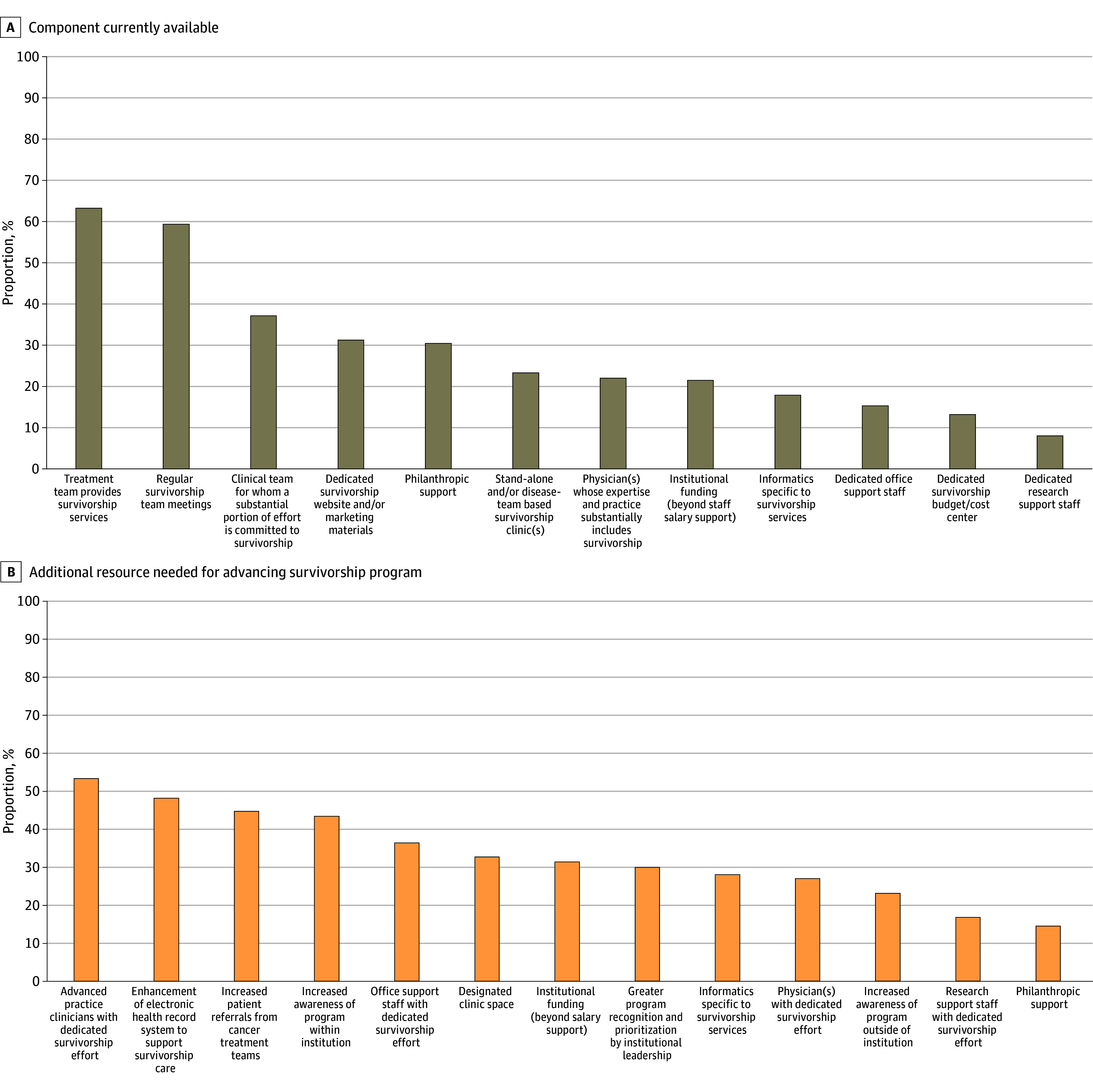
Existing Program Components and Additional Desired Resources Bars indicate the percentage of respondents in aggregate that endorsed each component as currently available (A) and each additional resource needed for advancing their survivorship program (B). For existing components, respondents selected all that applied; for resources needed, respondents selected the 5 most important. The total number of responses was 384.

Delivery of survivorship care by the same cancer treatment team was the most endorsed component (243 programs [63.3%]), followed by having regular team meetings (228 programs [59.4%]) (Figure 3A; eTable 2 in Supplement 1). Approximately one-third of programs had clinical team members with dedicated survivorship effort. Approximately one-fifth of programs had physicians with survivorship expertise, dedicated survivorship program funding, a survivorship database, dedicated office support staff, or a dedicated budget or cost center. Only 31 programs (8.1%) reported survivorship research staff. Across CoC program categories (eTable 2 in Supplement 1), Integrated Cancer Network Programs, Academic Comprehensive Cancer Programs, or NCI-Designated Comprehensive Cancer Programs endorsed the most program components. NCI-Designated Comprehensive Cancer Programs had the highest availability of specialized survivorship clinics (14 programs [73.7%]) and physicians with survivorship expertise (10 programs [52.6%]).

#### Resources Needed

As shown in Figure 3B and eTable 2 in Supplement 1, the 5 most commonly endorsed resources needed to advance their survivorship programs were APCs with dedicated survivorship effort (205 programs [53.4%], in contrast to 104 programs [27.1%] endorsing survivorship physicians), survivorship enhancements for the electronic health record system (185 programs [48.2%]), increased patient referrals from cancer treatment clinicians (172 programs [44.8%]), increased internal awareness of the program (167 programs [43.5%]), and survivorship office staff (140 programs [36.5%]). Needing dedicated institutional funding was endorsed twice as often as philanthropic support (121 programs [31.5%] vs 56 programs [14.6%]).

### Survivorship Program Perceptions

Table 2 and eTable 3 in Supplement 1 summarize program perceptions about the development, delivery, and impact of their survivorship care. Overall, 224 respondents (59.3%) reported having a survivorship program prior to the introduction of Survivorship Standard 4.8 in 2021. This was true most often among Free Standing Cancer Center programs (3 programs [100%]) and NCI-Designated Comprehensive Cancer programs (14 programs [73.7%]) and least often among Community Cancer programs (41 programs [50.0%]). In aggregate, most respondents definitely agreed (162 programs [43.1%]) or somewhat agreed (173 programs [46.0%]) that Survivorship Standard 4.8 facilitated initiation or advancement of their survivorship program. In estimating what proportion of eligible survivors receive available services, more than 80% of respondents endorsed some (229 respondents [60.7%]), few (77 respondents [20.4%]), or none (1 respondent [0.3%]). Institutions perceived the single most common barrier to receiving their survivorship services to be lack of referral (106 respondents [28.5%]) and survivors being unaware of services (86 respondents [23.1%]), in contrast to distance and insurance issues (both <10%) (Table 2). For patients who did receive services, programs perceived their impact as very beneficial (173 programs [46.6%]) or beneficial (145 programs [39.1%]).

**Table 2.** Institutional Perceptions About the Development, Delivery, and Impact of Survivorship Care

Perception^a^	Responses, No. (%)^b^
Institutions with a survivorship program before the CoC Survivorship Standard 4.8 requirement (n = 378)	224 (59.3)
Inclusion of Survivorship Standard 4.8 as part of CoC accreditation helped initiate or advance survivorship care at my institution (n = 376)	
Definitely agree	162 (43.1)
Somewhat agree	173 (46.0)
Somewhat disagree	28 (7.5)
Definitely disagree	13 (3.5)
Proportion of eligible patients receiving survivorship care (n = 377)	
Most	70 (18.6)
Some	229 (60.7)
Few	77 (20.4)
None	1 (0.3)
Most common reason patients do not receive survivorship care (n = 372)	
Lack of referral	106 (28.5)
Patient not aware of services	86 (23.1)
Do not know	60 (16.1)
Distance to services	28 (7.5)
Insurance barriers	14 (3.8)
Other^c^	78 (21.0)
Impact of survivorship care for patients who receive it (n = 371)	
Very beneficial	173 (46.6)
Beneficial	145 (39.1)
Somewhat beneficial	45 (12.1)
Minimally beneficial	8 (2.2)

Abbreviation: CoC, Commission on Cancer.

^a^ The full survey questions appear in eAppendix 2 in Supplement 1.

^b^ Total values may not sum to 384 due to item missingness.

^c^ Includes additional support not needed, lack of patient interest, lack of dedicated staff and time to provide services, lack of resources, patient lost to follow-up, patients underestimate their need for support, lack of a formalized clinic space or physician, lack of a formal structured program, no-show to appointments, patient does not see value, patients do not want to come back, geographic spread of population, financial burden, survivorship team not alerted by physician, lack of buy-in from health care practitioners, not easily available, and transportation.

## Discussion

This survey study characterized the prevalence and types of survivorship services available to survivors of adult-onset cancer across ACS CoC–accredited programs, a large consortium of facilities that spans the US, provides cancer care to three-quarters of all US patients with cancer, and includes both academic and community-based settings. Our findings indicate widespread availability of some survivorship services but also identify clear gaps, suggesting modifiable areas for intervention to improve their availability and uptake. This national benchmark for adult survivorship care enables assessing the impact of new approaches and tracking progress longitudinally. Although it is now recognized as an essential phase of cancer care, survivorship care remains largely underdeveloped, prompting its identification as a national priority and magnifying the importance of these findings.^29,30,31^

Several services were available to all survivors at nearly 90% of programs, notably screening for new or recurrent cancers and specialty referrals for managing late effects. Less available were cancer genetics counseling and, especially, fertility and sexual health services. Given the frequency of some pathogenic germline variants^32,33^ and the patient-reported importance of fertility and sexual health,^34,35,36,37^ these services require wider availability. Similarly, a small proportion of programs endorsed offering patient support groups, fitness programs, and educational series. A rich variety of allied survivorship services were reported by a minority of respondents; however, as many such services are not reimbursed by insurance, it remains challenging for institutions to offer them. Overall, these findings are similar to those of a 2024 study that assessed survivorship service availability as described on the CoC-accredited survivorship program websites.^38^ In that study by Anampa-Guzmán et al,^38^ cancer genetic counseling, fertility, and sexual health services also appeared to be underrepresented.

Treatment summaries and survivorship care plans provide relevant cancer-related health information to survivors and their health care practitioners.^39,40^ Recent studies have questioned the value of survivorship care plans due to uncertain benefits and the substantial resources they require.^41,42,43,44^ Thus, it was somewhat surprising that more than 90% of programs reported providing one or the other to all survivors (more frequent for treatment summaries) or certain subsets (more frequent for survivorship care plans). This could indicate that programs derive value from these documents or could represent the lasting impact of Survivorship Standard 3.3, in which distribution of survivorship care plans was the sole adherence metric until implementation of Survivorship Standard 4.8, which reflects a broader, programmatic emphasis.^19^

Two-thirds of programs reported that the cancer treatment team also provided survivorship care, whereas only one-third of programs offered separate, specialized survivorship clinics. This is quite different than pediatric oncology, where specialized late-effects clinics are widely implemented and considered the standard of care.^22^ Here, specialized survivorship clinics were more common among comprehensive cancer centers than community-based programs. The American Society of Clinical Oncology Survivorship Compendium recognizes disease- and treatment-specific survivorship care as a viable survivorship care model.^45,46^ Given the much higher prevalence of adult-onset cancer, this model might be more feasible and clinically appropriate than the specialized, resource-intensive approach common in pediatrics. Additional research is needed to determine optimal models of care following adult-onset cancer, accounting for cancer type, age, and risk profile.

Several programmatic themes emerged. Importantly, programs reported a lack of institutional support through dedicated salaries, marketing, philanthropy, informatics, cost centers, and office or research staff. Correspondingly, the most important additional resources needed for program advancement included dedicated APCs, enhanced information technology, and internal program visibility. Lower priority was given to having survivorship physicians, external marketing, research, and philanthropy. Consistent with these themes were the perceptions of survivorship program value and challenges. Despite nearly 90% of respondents endorsing benefit for survivors who receive their services, they indicated only a minority of patients receive them, with principal barriers being poor utilization by referring oncologists and low patient awareness. Collectively, these observations reveal a pressing need for institutions to prioritize survivorship care through investing in clinical and office staff, survivorship enhancements for electronic health record systems, patient-facing survivorship materials, and clear expectations for referral of patients to survivorship services. Research tracking the impacts of such initiatives is needed.

Many of the specific and thematic findings of our study validate the qualitative experiences of 8 selected survivorship programs in the US reported by Manne and Nekhlyudov.^47^ Across those programs, there was a similarly wide range of services offered and heterogeneity of clinical models. Although some programs used the electronic health record to improve communication among care staff, enhanced modules for facilitating treatment summaries appeared less available. Needed resources were similar, including sustainable funding sources, informatics for capturing outcomes data, and institutional engagement for developing credible business plans.^47^ In addition to providing quantitative data, important additional strengths of our study are leveraging the unique resource of CoC-accredited programs, which reflects cancer care across the US with an analytic sample of nearly 400 programs that captures the diversity of CoC-accredited institutions and practice settings. Furthermore, survey content mapped closely to CoC Survivorship Standard 4.8, currently the only US accreditation standard for adult-onset cancer survivorship.^16^

### Limitations

This study has some limitations. There is potential for participation bias related to the response rate. It is possible that a longer survey window could have increased this, although participation rates lower than 30% are not uncommon with clinical surveys.^48,49,50^ Furthermore, the need for higher participation rates to support face validity has been questioned when representativeness can be documented otherwise, as was the case in this study, in which the proportions of program categories among survey respondents were similar to those among CoC-accredited programs overall.^51^ This study did not evaluate access to services or their concordance with guidelines. Likewise, collection of survivorship program operational details was beyond the study scope and warrants future research.

## Conclusions

This survey study of CoC-accredited programs describes the current landscape of survivorship services available to patients with adult-onset cancer. Several conclusions can be drawn from the results. First, although survivorship care after adult-onset cancer is currently heterogenous, most participating programs offered many services aligned with the unmet needs of survivors. This provides an encouraging starting point for national efforts to advance survivorship care. Second, despite the multidisciplinary composition of survivorship program teams, opportunities for greater engagement of relevant professions (eg, nutrition, physical and occupational therapy, behavioral health) existed. Third, while some programs offered specialized survivorship clinics, more offered treatment team–based clinics, which may represent a more feasible and medically appropriate approach for survivors of adult-onset cancer. Fourth, for survivorship program advancement, institutions must invest resources to increase their capacity, visibility, and uptake. Fifth, survivorship care standards tied to performance metrics contributed to program development. With 90% of institutions endorsing CoC Survivorship Standard 4.8 as instrumental for initiating or developing their own programs, there is strong evidence to build on this and similar standards in the future. Initiatives that leverage successful programs to guide others in navigating the formidable challenges of program development may expedite the uniform delivery of high-quality and comprehensive survivorship care.

## Notes

Supplement 1. **eTable 1.** Survivorship Program Team Members by CoC Program Category **eTable 2.** Specialized Survivorship Clinics, Program Components, and Needed Resources by CoC Program Category **eTable 3.** Institutional Perceptions About the Development, Delivery, and Impact of Survivorship Care by CoC Program Category **eFigure.** Analytic Sample **eAppendix 1.** CoC Program-Related Definitions **eAppendix 2.** Survivorship Services Survey

Supplement 2.
Data Sharing Statement
