# Supplementary material for: Cancer Survivorship Care in the United States at Facilities Accredited by the Commission on Cancer
Source: JAMA Netw Open. 2024 Jul 3;7(7):e2418736. doi: 10.1001/jamanetworkopen.2024.18736 (PMC11222991; doi:10.1001/jamanetworkopen.2024.18736)
Supplement: Supplement 1. — eTable 1. Survivorship Program Team Members by CoC Program Category eTable 2. Specialized Survivorship Clinics, Program Components, and Needed Resources by CoC Program Category eTable 3. Institutional Perceptions About the Development, Delivery, and Impact of Survivorship Care by CoC Program Category eFigure. Analytic Sample eAppendix 1. CoC Program-Related Definitions eAppendix 2. Survivorship Services Survey [file jamanetwopen-e2418736-s001.pdf]

Supplemental Online Content

Stal J, Miller KA, Mullett TW, et al. Cancer survivorship care in the United States at facilities accredited by the Commission on Cancer. *JAMA Netw Open*. 2024;7(7):e2418736. doi:10.1001/jamanetworkopen.2024.18736

eTable 1. Survivorship Program Team Members by CoC program Category

eTable 2. Specialized Survivorship Clinics, Program Components, and Needed Resources by CoC Program Category

eTable 3. Institutional Perceptions About the Development, Delivery, and Impact of Survivorship Care by CoC Program Category

eFigure. Analytic Sample

eAppendix 1. CoC Program-Related Definitions

eAppendix 2. Survivorship Services Survey

This supplemental material has been provided by the authors to give readers additional information about their work.

© 2024 Stal J et al. *JAMA Network Open.*

eTable 1: Survivorship program team members by CoC program category

Team Member[#]	Total^	CoC Program Category[*]							
		Comprehensive Community Cancer Program	Community Cancer Program	Integrated Cancer Network Program	Academic Comprehensive Cancer Program	Hospital Associate Cancer Program	NCI Designated Comprehensive Cancer Program	Free Standing Cancer Center Program	NCI Designated Network
	n (%)	n (%)	n (%)	n (%)	n (%)	n (%)	n (%)	n (%)	n (%)
No. of programs responding	371 (96.6)	122	84	65	48	29	19	3	1
Nurse	334 (87.0)	109 (89.3)	77 (91.7)	54 (83.1)	40 (83.3)	25 (86.2)	15 (79.0)	3 (100.0)	1 (100.0)
Social worker	278 (72.4)	93 (76.2)	52 (61.9)	51 (78.5)	33 (68.8)	21 (72.4)	13 (68.4)	3 (100.0)	1 (100.0)
Coordinator	275 (71.6)	88 (72.1)	59 (70.2)	51 (78.5)	32 (66.7)	20 (69.0)	12 (63.2)	3 (100.0)	1 (100.0)
Advanced practice provider (nurse practitioner, physician assistant)	252 (65.6)	74 (60.7)	43 (51.2)	54 (83.1)	38 (79.2)	18 (62.1)	15 (79.0)	3 (100.0)	1 (100.0)
Nutritionist	250 (65.1)	81 (66.4)	50 (59.5)	39 (60.0)	34 (70.8)	22 (75.9)	10 (52.6)	3 (100.0)	1 (100.0)
Physician	243 (63.3)	69 (56.6)	45 (53.6)	46 (70.8)	39 (81.3)	16 (55.2)	14 (73.7)	3 (100.0)	1 (100.0)
Physical therapist	180 (46.9)	61 (50.0)	39 (46.4)	25 (38.5)	22 (45.8)	14 (48.3)	9 (47.4)	0	0
Occupational therapist	87 (22.7)	29 (23.8)	12 (14.3)	15 (23.1)	10 (20.8)	9 (31.0)	6 (31.6)	0	0
Other allied health professionals[@]	148 (38.5)	51 (41.8)	33 (39.3)	18 (27.7)	23 (47.9)	6 (20.7)	9 (47.4)	1 (33.3)	0

[*]Column percents

^Denominator for Total column = 384. Program category responses may not sum to total values due to item missingness.

[#]Participants could select more than one response

[@]Acupuncturist, Administrator, Art and Music Therapist, Massage Therapist, Cancer Center Manager, Cancer Program Coordinator, Cancer Program Associate, Cancer Registrar, Case Manager, Certified Community Health Worker, Chaplain, Clinical Trial Nurse, Community Partner, Director of Rehabilitation, Education Coordinator, Exercise Physiologist, Financial Counselor/Navigator, Integrative Health Specialist, Life Coach, Lymphedema Therapist, Medical Assistant, Mindfulness Counselor, Oncology Counselor, Palliative Care, Patient Navigator, Pharmacist, Psychology/Behavioral Health Counselor, Radiation Therapist, Researcher, Respiratory Therapist, Sexual Health Counselor, Speech-Language Pathologist, Speech Therapist, Spiritual Care, Tobacco Cessation Coordinator, Tumor Registry, Wellness Specialist and Yoga Instructor.

© 2024 Stal J et al. *JAMA Network Open.*

eTable 2: Specialized survivorship clinics, program components, and needed resources by CoC program category

		CoC Program Category*							
	**Total^**	Comprehensive Community Cancer Program	Community Cancer Program	Integrated Cancer Network Program	Academic Comprehensive Cancer Program	Hospital Associate Cancer Program	NCI Designated Comprehensive Cancer Program	Free Standing Cancer Center Program	NCI Designated Network
	n (%)	n (%)	n (%)	n (%)	n (%)	n (%)	n (%)	n (%)	n (%)
No. of programs responding	371 (96.6)	122	84	65	48	29	19	3	1
**Specialized Survivorship Clinic**									
Have a specialized survivorship clinic and/or survivorship clinics dedicated to providing survivorship care to adult-onset cancer patients	120 (31.3)**	27	15	27	21	10	14	0	0
**Survivor population served%#**									
All survivors	58 (48.3)	17 (63.0)	7 (46.7)	13 (48.2)	10 (47.6)	4 (40.0)	4 (28.6)	.	.
Selected survivor groups (e.g., by cancer type, treatment modality, age, etc.)	57 (47.5)	10 (37.0)	6 (40.0)	14 (51.9)	11 (52.4)	6 (60.0)	8 (57.1)	.	.
Other@	11 (9.2)	2 (7.4)	2 (13.3)	0	2 (9.5)	0	4 (28.6)	.	.
**Survivorship Program Components**									
**Existing program components#**									
Treatment team provides survivorship services	243 (63.3)	80 (65.6)	44 (52.4)	49 (75.4)	35 (72.9)	20 (69.0)	6 (31.6)	2 (66.7)	0
Regular survivorship team meetings	228 (59.4)	71 (58.2)	43 (51.2)	45 (69.2)	30 (62.5)	17 (58.6)	12 (63.2)	2 (66.7)	1 (100.0)
Clinical team for whom a substantial portion of effort is committed to survivorship	143 (37.2)	40 (32.8)	24 (28.6)	33 (50.8)	21 (43.8)	10 (34.5)	8 (42.1)	1 (33.3)	0
Dedicated survivorship website and/or marketing materials	120 (31.3)	28 (23.0)	25 (29.8)	31 (47.7)	17 (35.4)	9 (31.0)	8 (42.1)	1 (33.3)	0
Philanthropic support	117 (30.5)	28 (23.0)	29 (34.5)	23 (35.4)	16 (33.3)	7 (24.1)	6 (31.6)	2 (66.7)	1 (100.0)
Stand-alone and/or disease-team based survivorship clinic(s)	90 (23.4)	18 (14.8)	6 (7.1)	21 (32.3)	19 (39.6)	7 (24.1)	14 (73.7)	0	0
Physician(s) whose expertise and practice substantially includes survivorship	85 (22.1)	14 (11.5)	16 (19.1)	12 (18.5)	21 (43.8)	8 (27.6)	10 (52.6)	1 (33.3)	0
Institutional funding (e.g., website development, event or program funding)	83 (21.6)	15 (12.3)	19 (22.6)	25 (38.5)	11 (22.9)	4 (13.8)	6 (31.6)	1 (33.3)	1 (100.0)
Database dedicated to following survivors	69 (18.0)	20 (16.4)	11 (13.1)	10 (15.4)	13 (27.1)	8 (27.6)	3 (15.8)	1 (33.3)	0
Dedicated office support staff (e.g., office assistant, project assistant, etc.)	59 (15.4)	10 (8.2)	5 (6.0)	20 (30.8)	11 (22.9)	5 (17.2)	6 (31.6)	0	0
Dedicated survivorship budget/cost center	51 (13.3)	13 (10.7)	5 (6.0)	16 (24.6)	6 (12.5)	3 (10.3)	5 (26.3)	2 (66.7)	0

© 2024 Stal J et al. *JAMA Network Open.*

Clinical research staff dedicated to survivorship program team	31 (8.1)	5 (4.1)	4 (4.8)	7 (10.8)	7 (14.6)	4 (13.8)	3 (15.8)	1 (33.3)	0
**Additional resources not already available that would be most important for helping survivorship program achieve its goals[&]**									
Advanced practice providers (nurse practitioner, physician assistant) with dedicated survivorship effort	205 (53.4)	69 (56.6)	51 (60.7)	26 (40.0)	21 (43.8)	17 (58.6)	10 (52.6)	2 (66.7)	1 (100.0)
Enhancement of electronic health record system to support survivorship care	185 (48.2)	57 (46.7)	42 (50.0)	39 (60.0)	19 (39.6)	11 (37.9)	8 (42.1)	1 (33.3)	0
Increased patient referrals from cancer treatment teams	172 (44.8)	57 (46.7)	36 (42.9)	29 (44.6)	23 (47.9)	10 (34.5)	10 (52.6)	1 (33.3)	0
Increased awareness of program within institution	167 (43.5)	47 (38.5)	43 (51.2)	29 (44.6)	22 (45.8)	16 (55.2)	6 (31.6)	0	0
Office support staff with dedicated survivorship effort (e.g., office assistant, project assistant)	140 (36.5)	40 (32.8)	31 (36.9)	16 (24.6)	26 (54.2)	8 (27.6)	10 (52.6)	2 (66.7)	1 (100.0)
Designated clinic space	126 (32.8)	44 (36.1)	29 (34.5)	19 (29.2)	15 (31.3)	10 (34.5)	5 (26.3)	0	1 (100.0)
Institutional funding (e.g., website development, event or program funding)	121 (31.5)	41 (33.6)	28 (33.3)	15 (23.1)	15 (31.3)	14 (48.3)	2 (10.5)	2 (66.7)	0
Greater program recognition and prioritization by institutional leadership	115 (30.0)	35 (28.7)	23 (27.4)	14 (21.5)	19 (39.6)	11 (37.9)	8 (42.1)	0	0
Informatics specific to survivorship services (e.g., clinical and/or research database)	108 (28.1)	36 (29.5)	16 (19.1)	28 (43.1)	9 (18.8)	7 (24.1)	9 (47.4)	0	0
Physicians(s) with dedicated survivorship effort	104 (27.1)	27 (22.1)	19 (22.6)	31 (47.7)	11 (22.9)	4 (13.8)	7 (36.8)	1 (33.3)	1 (100.0)
Increased awareness of program outside of institution (e.g., external marketing)	89 (23.2)	24 (19.7)	17 (20.2)	22 (33.9)	9 (18.8)	10 (34.5)	3 (15.8)	0	0
Research support staff with dedicated survivorship effort (e.g., research assistant, clinical research coordinator, etc.)	65 (16.9)	17 (13.9)	16 (19.1)	4 (6.2)	16 (33.3)	2 (6.9)	5 (26.3)	0	1 (100.0)
Philanthropic support	56 (14.6)	23 (18.9)	9 (10.7)	10 (15.4)	6 (12.5)	3 (10.3)	4 (21.1)	0	0

[*]Column percents

^Denominator for Total column = 384. Program category responses may not sum to total values due to item missingness.

**6 programs that did not specify program category endorsed having a survivorship clinic.

[%]Percentages are among programs reporting a specialized survivorship clinic.

[#]Participants could select more than one response

[@]Includes: Any survivor but by referral/need, cancer risk clinics, general survivorship clinic, small general survivorship clinic

[&]Only 5 responses were allowed as participants were asked to select the five most important

© 2024 Stal J et al. *JAMA Network Open.*

eTable 3: Institutional perceptions about the development, delivery, and impact of survivorship care by CoC program category

	**CoC Program Category**[*^]							
	Comprehensive Community Cancer Program	Community Cancer Program	Integrated Cancer Network Program	Academic Comprehensive Cancer Program	Hospital Associate Cancer Program	NCI Designated Comprehensive Cancer Program	Free Standing Cancer Center Program	NCI Designated Network
	n (%)	n (%)	n (%)	n (%)	n (%)	n (%)	n (%)	n (%)
No. of programs responding	122	84	65	48	29	19	3	1
**Institutions with a survivorship program before the CoC Survivorship Standard 4.8 requirement (2021)**								
	80 (66.1)	41 (50.0)	33 (50.8)	29 (61.7)	17 (60.7)	14 (73.7)	3 (100.0)	.
**Inclusion of Standard 4.8 as part of CoC accreditation has helped initiate and/or advance survivorship care at my institution.**[a]								
Definitely agree	43 (35.8)	34 (41.5)	34 (52.3)	24 (52.2)	12 (41.4)	6 (33.3)	1 (33.3)	0
Somewhat agree	57 (47.5)	39 (47.6)	29 (44.6)	17 (37.0)	15 (51.7)	9 (50.0)	2 (66.7)	1 (100.0)
Somewhat disagree	12 (10.0)	7 (8.5)	1 (1.5)	4 (8.7)	1 (3.5)	3 (16.7)	0	0
Definitely disagree	8 (6.7)	2 (2.4)	1 (1.5)	1 (2.2)	1 (3.5)	0	0	0
**For patients who are eligible for existing survivorship services at your institution, what proportion receive them?**								
Most	16 (13.3)	23 (28.1)	9 (14.1)	9 (19.6)	11 (37.9)	1 (5.3)	1 (33.3)	0
Some	75 (62.5)	46 (56.1)	38 (59.4)	30 (65.2)	15 (51.7)	12 (63.2)	1 (33.3)	1 (100.0)
Few	29 (24.2)	12 (14.6)	17 (26.6)	7 (15.2)	3 (10.3)	6 (31.6)	1 (33.3)	0
None	0	1 (1.2)	0	0	0	0	0	0
**For patients who do not receive survivorship services at your institution, what do you think is the most common reason?**								
Lack of referral	33 (28.2)	21 (25.9)	17 (26.6)	17 (37.0)	5 (17.9)	9 (47.4)	1 (33.3)	0
Patient not aware of services	26 (22.2)	20 (24.7)	20 (31.3)	6 (13.0)	6 (21.4)	4 (21.1)	1 (33.3)	0
Do not know	18 (15.4)	16 (19.8)	9 (14.1)	8 (17.4)	6 (21.4)	1 (5.3)	0	0
Distance to services	8 (6.8)	8 (9.9)	3 (4.7)	4 (8.7)	2 (7.1)	1 (5.3)	1 (33.3)	1 (100.0)
Insurance barriers	6 (5.1)	3 (3.7)	2 (3.1)	3 (6.5)	0	0	0	0
Other[#]	26 (22.2)	13 (16.1)	13 (20.3)	8 (17.4)	9 (32.1)	4 (21.1)	0	0

© 2024 Stal J et al. *JAMA Network Open.*

eTable 3, continued

	CoC Program Category[*]							
	Comprehensive Community Cancer Program	Community Cancer Program	Integrated Cancer Network Program	Academic Comprehensive Cancer Program	Hospital Associate Cancer Program	NCI Designated Comprehensive Cancer Program	Free Standing Cancer Center Program	NCI Designated Network
	n (%)	n (%)	n (%)	n (%)	n (%)	n (%)	n (%)	n (%)
No. of programs responding	122	84	65	48	29	19	3	1
**For patients who do receive survivorship care at your institution, what impact do you feel it has for them?**								
Very beneficial	48 (40.3)	30 (37.0)	26 (41.3)	26 (57.8)	14 (51.9)	16 (84.2)	3 (100.0)	1 (100.0)
Beneficial	54 (45.4)	34 (42.0)	27 (42.9)	15 (33.3)	12 (44.4)	2 (10.5)	0	0
Somewhat beneficial	12 (10.1)	15 (18.5)	9 (14.3)	4 (8.9)	1 (3.7)	1 (5.3)	0	0
Minimally beneficial	5 (4.2)	2 (2.5)	1 (1.6)	0	0	0	0	0

[*]Column percents
[^]Program category responses may not sum to total due to item missingness

[#]Includes: Additional support not needed, lack of patient interest, lack of dedicated staff and time to provide services, lack of resources, patient lost to follow-up, patients underestimate their need for support, lack of a formalized clinic space or physician, lack of a formal structured program, no show to appointments, patient does not see value, patients do not want to come back, geographic spread of population, financial burden, survivorship team not alerted by physician, lack of buy in from providers, not easily available, transportation

© 2024 Stal J et al. *JAMA Network Open.*

eFigure: Analytic Sample

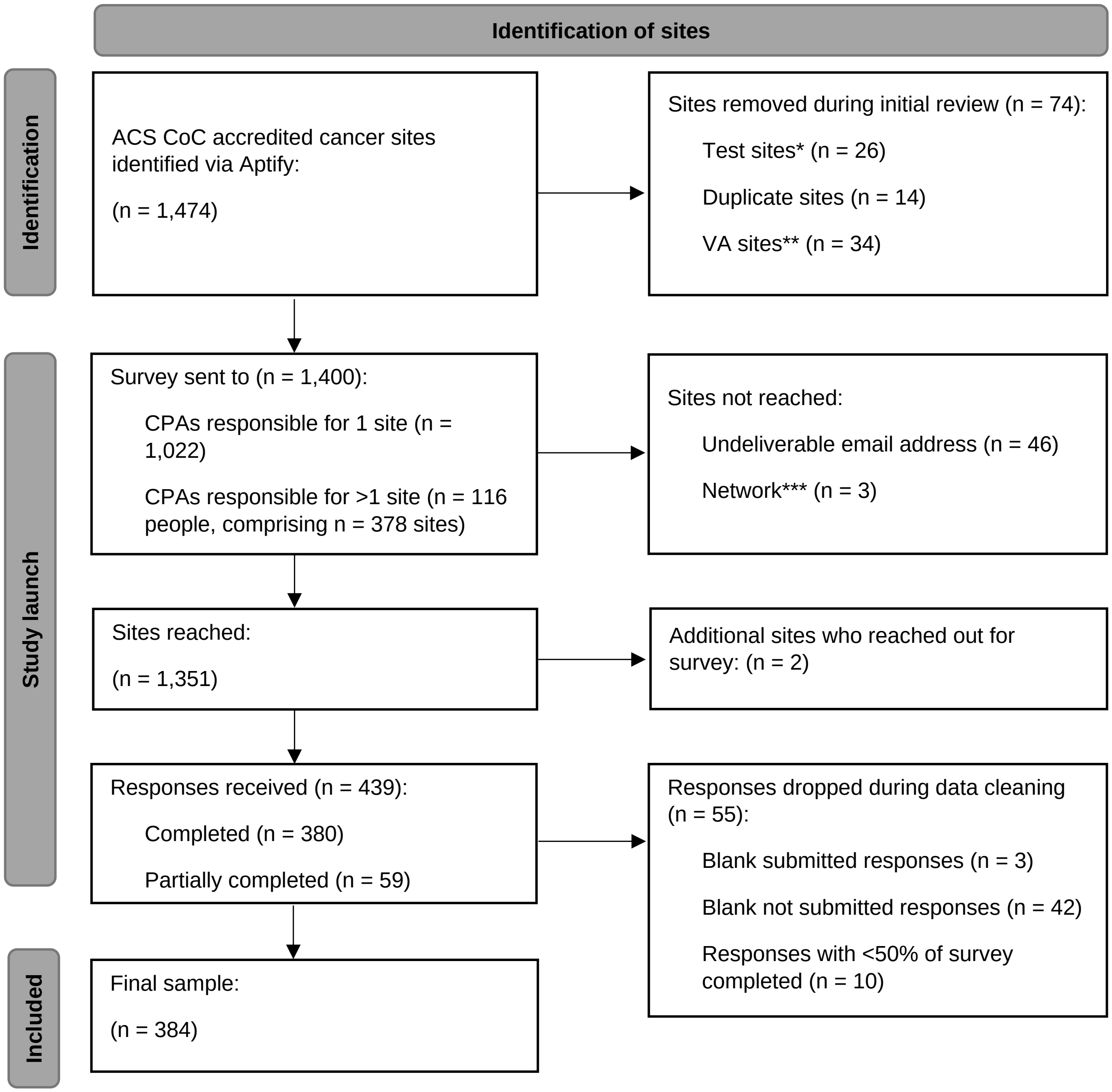

eFigure. Analytic Sample. *Test (dummy) sites are included in Aptify for research purposes and are not actual facilities. **VA sites were excluded due to data usage restrictions. ***Facilities belonging to an organization that owns a group of facilities that offer integrated and comprehensive cancer care services and is overseen by a centralized governance structure/board and CEO. ACS CoC=American College of Surgeons Commission on Cancer; VA=Veterans Affairs; CPA=Cancer Program Administrator.

© 2024 Stal J et al. *JAMA Network Open.*

eAppendix 1: CoC Program-related Definitions

Title	Definition
Accredited	Awarded when the program has completed a site visit and demonstrated full compliance with all applicable standards and has provided all required documentation to support compliance: Program appears on Find an Accredited Program website; Certificate of accreditation awarded.
**American College of Surgeons Institutional Program**	
Academic Comprehensive Cancer Program (ACAD)	The facility participates in postgraduate medical education in at least four program areas, including internal medicine and general surgery. The facility accessions more than 500 newly diagnosed cancer cases each year.
Community Cancer Program (CCP)	The facility accessions more than 100 but fewer than 500 newly diagnosed cancer cases each year.
Comprehensive Community Cancer Program (CCCP)	The facility accessions 500 or more newly diagnosed cancer cases each year.
Free Standing Cancer Center Program (FCCP)	The facility is a nonhospital-based program and offers at least one cancer-related treatment modality. The full range of diagnostic and treatment services is available by referral. Referral to CoC-accredited cancer program(s) is preferred. There is no minimum caseload requirement for this category.
Hospital Associate Cancer Program (HACP)	The facility accessions 100 or fewer newly diagnosed cancer cases each year and has a limited range of diagnostic and treatment services available on-site. Other services are available by referral. Clinical research is not required.
Integrated Network Cancer Program (INCP)	Facilities belonging to an organization that owns a group of facilities that offer integrated and comprehensive cancer care services and is overseen by a centralized governance structure/board and CEO.
NCI Designated Comprehensive Cancer Program (NCIP)	The facility secures a National Cancer Institute (NCI) peer-reviewed Cancer Center Support Grant and is designated a Comprehensive Cancer Center by the NCI. A full range of diagnostic and treatment services and staff physicians are available. Participation in the training of resident physicians is optional, and there is no minimum caseload requirement for this category.
NCI Designated Network (NCIN)	Facilities belonging to an organization that owns a group of facilities that offer integrated and comprehensive cancer care services and is overseen by a centralized governance structure/board and CEO. Additionally, the facilities secure a National Cancer Institute (NCI) peer-reviewed Cancer Center Support Grant and are designated a Comprehensive Cancer Center Consortium by the NCI. To be included in the NCIN, all facilities must be included within the NCI grant.
**Role of Person Completing Survey**	
Cancer Committee Chair	Physician of any specialty, selected according to facility rules and/or bylaws; can also represent one of the required physician specialties.
Cancer Liaison Chair	Physician of any specialty who is an active member of the medical staff and is selected and appointed by the Cancer Committee.
Cancer Program Administrator	Responsible for the administrative oversight and has budget authority for the cancer program.
Certified Tumor Registrar	CTRs apply knowledge obtained from formal education and work experience to correctly interpret and code cancer diagnosis, stage, treatment, and outcomes information for each case that is seen at the Commission on Cancer (CoC)-accredited program that meets CoC reporting requirements.
Survivorship Program Coordinator	Responsible for overseeing Standard 4.8: Survivorship Program; a physician, physician assistant, advanced practice nurse, nurse, social worker (Oncology Social Worker certified by the Board of Oncology Social Work [OSW-C] preferred), nurse navigator, or therapist or other licensed health care professional is selected to fill this role.

© 2024 Stal J et al. *JAMA Network Open.*

eAppendix 2: Survivorship Services Survey

Thank you for participating in this survey sponsored by the American College of Surgeons Commission on Cancer (CoC)!

The objective of this survey is to learn more about existing cancer survivorship programs for patients treated for adult-onset cancer, including personnel, available services, and resources needed to enhance survivorship care provided as part of these programs. The survey should take approximately 20 minutes or less to complete. If necessary, you will be able to save your work and return later to complete the survey.

In responding to these questions, please keep in mind the following:

Instructions for survey completion:

- The respondent should be the person most knowledgeable about your survivorship program. If there is someone other than you who is better positioned to respond, please forward this survey to that person.
- It is understood the respondent may need to confer with other personnel for some items.
- Please submit only one response per institution. If you are a member of an Integrated Network Cancer Program (INCP) or a NCI-Designated Network Cancer Program (NCIN), the survey must be filled out at the level of each individual hospital (e.g., the "parent" and "child" hospital).
- Please note: Pediatric Cancer Programs and Veterans Affairs Cancer Programs are excluded from this study.
- Please answer all questions as they relate to CoC Survivorship Program Standard 4.8.

Key definitions for this survey:

- Survivorship Program: A program directed at meeting the needs of cancer patients treated with curative intent. For purposes of this survey, this refers to patients with adult-onset cancer.
- Cancer Survivor: A cancer patient treated with curative intent who has completed the "acute phase" of conventional therapy (i.e., cytotoxic chemotherapy, radiation therapy, cancer-directed surgery). For purposes of this survey, some of these patients may still be receiving an extended course or "chronic phase" of non-cytotoxic therapy to prevent recurrence/progression (e.g., aromatase inhibitors, immunomodulators, etc.).

SURVEY

1. Institutional Characteristics

What is your American College of Surgeons institutional program category? (Select one)

- Academic Comprehensive Cancer Program
- Community Cancer Program
- Comprehensive Community Cancer Program
- Hospital Associate Cancer Program
- Free Standing Cancer Center Program
- Integrated Cancer Network Program
- NCI Designated Network
- NCI Designated Comprehensive Cancer Program

What was your analytic case load for calendar year 2021? (Select one)

- 0-99
- 100-249
- 250-499
- 500-999
- 1,000-4,999
- 5,000+

What is the geographic region where your institution is located? (Select one)

- New England (CT, MA, ME, NH, RI, VT)
- Middle Atlantic (NJ, NY, PA)
- South Atlantic (DC, DE, FL, GA, MD, NC, SC, VA, WV)
- East North Central (IL, IN, MI, OH, WI)
- East South Central (AL, KY, MS, TN)
- West North Central (IA, KS, MN, MO, ND, NE, SD)
- West South Central (AR, LA, OK, TX)

© 2024 Stal J et al. *JAMA Network Open.*

- Mountain (AZ, CO, ID, MT, NM, NV, UT, WY)
- Pacific (AK, CA, HI, OR, WA)

What is the role of the person(s) completing this survey? (Select all that apply)
- Cancer Committee Chair
- Cancer Program Administrator
- Cancer Liaison Physician
- Cancer Registrar
- Survivorship Program Coordinator
- Other (please specify) __________

2. Survivorship Program Team

Please indicate what professionals are members of your survivorship program* team responsible for implementing Survivorship Program Standard 4.8. (Select all that apply)
- Coordinator(s)
- Physicians(s)
- Advanced practice provider(s) (nurse practitioner, physician assistant)
- Nurse(s)
- Social worker(s)
- Nutritionist(s)
- Physical therapist(s)
- Occupational therapist(s)
- Other allied health professional(s) (please specify) __________

**Survivorship Program: A program directed at meeting the needs of cancer patients treated with curative intent. For purposes of this survey, this refers to patients with adult-onset cancer.*

3. Survivorship Program Services*

Please indicate the current availability of each of the survivorship services listed.

Available services refer to on-site (the accredited facility or off-campus locations that are owned or part of the hospital licensure) or available through referral (services provided to the patient at a facility or physician office external to the cancer program).

	**Available to all survivors (on site or through referral)****	**Available to selected survivor groups (e.g., who meet eligibility criteria)****	**Not available**	**Do not know**
Treatment summaries				
Survivorship care plans				
Rehabilitation services (e.g., physical therapy, occupational therapy)				
Nutritional services (e.g., consultation with dietitian, provision of cancer-specific nutritional info)				
Sexual health services (e.g., pelvic floor therapy, erectile dysfunction, sex therapy)				
Fertility consultation and management (e.g., fertility assessment, preservation, restoration)				
Screening programs for cancer recurrence				

Screening for new cancers				
Seminars for patients (e.g., webinars, speakers)				
Patient support groups and services				
Psychological support and psychiatric services				
Formalized referrals to medical or surgical specialists for assessment or care of adverse treatment sequelae (e.g., cardiology, endocrinology, pulmonology)				
Financial support services				
Physical activity/fitness programs				
Cancer genetics counseling				
Other (please specify _________________)				

**Survivorship Program: A program directed at meeting the needs of cancer patients treated with curative intent. For purposes of this survey, this refers to patients with adult-onset cancer.*
***Cancer Survivor: A cancer patient treated with curative intent who has completed the "acute phase" of conventional therapy (i.e., cytotoxic chemotherapy, radiation therapy, cancer-directed surgery). For purposes of this survey, some of these patients may still be receiving an extended course or "chronic phase" of non-cytotoxic therapy to prevent recurrence/progression (e.g., aromatase inhibitors, immunomodulators, etc.).*

4. Specialized Survivorship Clinic

Do you have a specialized survivorship clinic and/or survivorship clinics dedicated to providing survivorship care to adult-onset cancer patients?

- Yes *(Proceed to next item)*
- No *(Skip to section 5)*

Please indicate the survivor population seen in the survivorship clinic(s). (Select all that apply)*

- All survivors
- Selected survivor groups (e.g., by cancer type, treatment modality, age, etc.)
- Other (please specify) __________

**Cancer Survivor: A cancer patient treated with curative intent who has completed the "acute phase" of conventional therapy (i.e., cytotoxic chemotherapy, radiation therapy, cancer-directed surgery). For purposes of this survey, some of these patients may still be receiving an extended course or "chronic phase" of non-cytotoxic therapy to prevent recurrence/progression (e.g., aromatase inhibitors, immunomodulators, etc.).*

5. Survivorship Programmatic Features

Please indicate features available in your survivorship program. (Select all that apply)

- Stand-alone and/or disease-team based survivorship clinic(s)
- Physician(s) whose expertise and practice substantially include survivorship
- Clinical team for whom a substantial portion of effort is committed to survivorship
- Treatment team provides survivorship services
- Regular survivorship team meetings
- Dedicated survivorship budget/cost center
- Dedicated office support staff (e.g., office assistant, project assistant, etc.)
- Clinical research staff dedicated to survivorship program team
- Dedicated survivorship website and/or marketing materials
- Database dedicated to following survivors
- Institutional funding (e.g., website development, event or program funding)
- Philanthropic support

© 2024 Stal J et al. *JAMA Network Open.*

6. Additional Resources Needed

What additional resources not already available at your institution do you feel would be most important for helping your survivorship program achieve its goals? (Select the 5 most important)

- Physician(s) with dedicated survivorship effort
- Advance practice providers (nurse practitioner, physician assistant) with dedicated survivorship effort
- Office support staff with dedicated survivorship effort (e.g., office assistant, project assistant, etc.)
- Research support staff with dedicated survivorship effort (e.g., research assistant, clinical research coordinator, etc.)
- Increased patient referrals from cancer treatment teams
- Designated clinic space
- Enhancement of electronic health record system to support survivorship care
- Greater program recognition and prioritization by institutional leadership
- Increased awareness of program within institution
- Increased awareness of program outside of institution (e.g., external marketing)
- Informatics specific to survivorship services (e.g., clinical and/or research database)
- Institutional funding (e.g., website development, event or program funding)
- Philanthropic support

7. Program Perceptions

Beyond providing survivorship care plans, did your institution have a survivorship program before the CoC Survivorship Program Standard 4.8 requirement (2021)?

- Yes
- No

Please rate your agreement with this statement:
The inclusion of Survivorship Program Standard 4.8 as part of the CoC accreditation has helped initiate and/or advance survivorship care at my institution. (Select one)

- Definitely agree
- Somewhat agree
- Somewhat disagree
- Definitely disagree

For patients who are eligible for eligible for existing survivorship services at your institution, what proportion do you estimate receive them?

- Most
- Some
- Few
- None

For patients who do not receive survivorship services that are available at your institution, what do you think is the most common reason? (Select one)

- Distance to services
- Lack of referral
- Patient not aware of services
- Insurance barriers
- Do not know
- Other (please specify) __________

For patients who do receive survivorship care at your institution, what impact do you feel it has for them?

- Very beneficial
- Beneficial
- Somewhat beneficial
- Minimally beneficial

© 2024 Stal J et al. *JAMA Network Open.*
