# Supplementary material for: Cancer Survivorship Care in the United States at Facilities Accredited by the Commission on Cancer
Source: JAMA Netw Open. 2024 Jul 3;7(7):e2418736. doi: 10.1001/jamanetworkopen.2024.18736 (PMC11222991; doi:10.1001/jamanetworkopen.2024.18736)
Supplement: Supplement 2. — Data Sharing Statement [file jamanetwopen-e2418736-s002.pdf]

# Data Sharing Statement

Stal. Cancer Survivorship Care in the United States at Facilities Accredited by the Commission on Cancer. *JAMA Netw Open*. Published July 03, 2024.
doi:10.1001/jamanetworkopen.2024.18736

**Data**
**Data available:** No

**Additional Information**
**Explanation for why data not available:** Data are not available for sharing in accordance with American College of Surgeons policy.
